# Targeting stroke risk and improving outcomes in patients with atrial fibrillation in Latin America: Identificando o risco de acidente vascular cerebral e melhorando desfechos em pacientes com fibrilação atrial na América Latina

**DOI:** 10.1590/1516-3180.2015.0222110716

**Published:** 2016-06-03

**Authors:** Bruce Stambler, Fernando Scazzuso

**Affiliations:** I MD. Director, Cardiac Arrhythmia Research and Education, Piedmont Heart Institute, Atlanta, United States.; II MD. Chief, Department of Electrophysiology, Instituto Cardiovascular de Buenos Aires (ICBA), Buenos Aires, Argentina.

**Keywords:** Stroke, Atrial fibrillation, Anticoagulants, Warfarin, Risk factors, Acidente vascular cerebral, Fibrilação atrial, Anticoagulantes, Varfarina, Fatores de risco

## Abstract

**CONTEXT AND OBJECTIVE:**

To examine stroke risk factors, including atrial fibrillation, management and prevention, and stroke outcomes across Latin America.

**DESIGN AND SETTING:**

Narrative review conducted at Piedmont Heart Institute, United States.

**METHODS:**

The PubMed, Embase and Cochrane databases were searched for stroke AND "Latin America" AND epidemiology (between January 2009 and March 2015). Further studies in the SciELO, World Health Organization and Pan-American Health Organization databases were used to address specific points.

**RESULTS:**

Countries categorized as low or middle-income nations by the World Bank, which includes most of Latin America, account for two-thirds of all strokes. Globally, fewer than half of patients (median treatment level: 43.9%) with atrial fibrillation receive adequate anticoagulation to reduce stroke risk, which correlates with data from Latin America, where 46% of outpatients did not receive guideline-compliant anticoagulation, ranging from 41.8% in Brazil to 54.8% in Colombia.

**CONCLUSIONS:**

Atrial fibrillation-related stroke carries a heavy burden. Non-vitamin K antagonist oral anti-coagulants provide options for reducing the risk of atrial fibrillation-related stroke. However, cost-effectiveness comparisons with warfarin are warranted before observational health-economics study results can be applied clinically. Initiatives to remedy inequalities and improve access to care across Latin America should accompany risk factor modification and guideline-based prevention.

## RESUMO

**CONTEXTO E OBJETIVO:**

Examinar os fatores de risco para acidente vascular cerebral (derrame), incluindo fibrilação atrial, manejo e prevenção, e desfechos do derrame na América Latina.

**TIPO DE ESTUDO E LOCAL:**

Revisão narrativa da literatura, realizada no Instituto do Coração Piedmont, Estados Unidos.

**MÉTODOS:**

Os termos "derrame" E "América Latina" E epidemiologia (entre janeiro de 2009 e março de 2015) foram buscados nas bases de dados PubMed, Embase e Cochrane. Estudos adicionais nas bases de SciELO, Organização Mundial da Saúde e Organização Pan-Americana de Saúde foram utilizados para discutir pontos específicos.

**RESULTADOS:**

Os países classificados como de baixa ou média renda pelo Banco Mundial, que incluem a maior parte da América Latina, representam dois terços de todos os derrames. Mundialmente, menos da metade dos pacientes (nível de tratamento mediano: 43,9%) com fibrilação atrial recebe anticoagulação adequada para reduzir o risco de derrame, o que correlaciona com os dados da América Latina, onde 46% dos pacientes ambulatoriais não receberam anticoagulação conforme as diretrizes, variando de 41,8% no Brasil a 54,8% na Colômbia.

**CONCLUSÕES:**

Derrames associados à fibrilação atrial impõem um ônus significativo. Anticoagulantes orais antagônicos sem vitamina K oferecem opções de redução do risco de derrames associados a fibrilação atrial. No entanto, comparações do custo-benefício com varfarina são justificáveis antes da aplicação clínica de resultados dos estudos observacionais relativos à economia da saúde. Iniciativas para solucionar diferenças e melhorar o acesso aos cuidados médicos na América Latina devem acompanhar a modificação dos fatores de risco e a prevenção baseada em orientações.

## INTRODUCTION

Stroke is a serious challenge in Latin America and throughout the world. The Global Burden of Disease (GBD) study estimated that in 2013 there were approximately 26 million stroke survivors worldwide, 71% of whom had experienced ischemic strokes.^1^ In the same year, 10.3 million people experienced new strokes (67% consisting of ischemic stroke) and 6.5 million people died from stroke (51% consisting of ischemic stroke).^1^ Two-thirds of all strokes occur in low and middle-income countries,^2^^,^^3^ as categorized by the World Bank, which include most of Latin America.^3^^,^^4^^,^^5^ Continuing studies will help to clarify the complexities of stroke epidemiology.^6^^,^^7^^,^^8^^,^^9^According to the most recent figures available from the Pan-American Health Organization (PAHO) for the Americas region, cardiovascular diseases accounted for 1.6 million deaths in 2012, of which 22% were due to cerebrovascular diseases (ahead of heart failure and hypertension, each with 9% of the total).^10^ A similar pattern was observed throughout the region, with some variations. For example, 31% of cardiovascular deaths in Brazil were attributed to cerebrovascular diseases.^10^ The GBD investigators noted that age-standardized stroke incidence, mortality, prevalence and stroke-related disability declined from 1990 to 2013.^1^ However, over the same period, the absolute number of people affected by stroke increased considerably across the globe, suggesting that the worldwide stroke burden continues to increase due to population growth and aging.^1^

**Figure 1:**
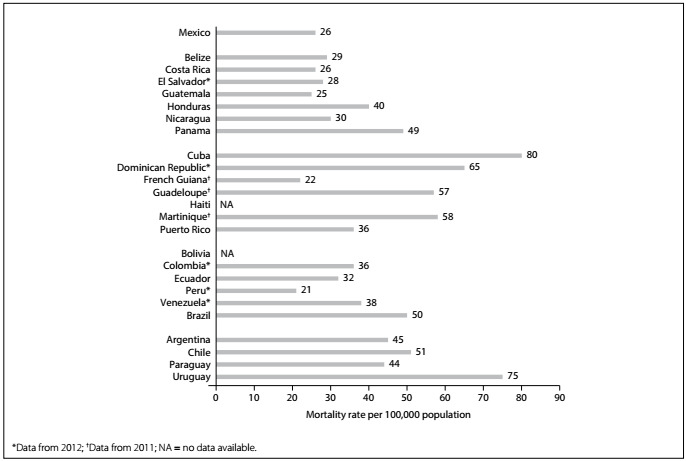
Stroke mortality (per 100,000 population per year) in Latin America by country, 2013.^10^

Although most of the burden of stroke is borne by low- and middle-income countries, stroke incidence rates have fallen concomitantly with reductions in risk factors associated with stroke therapies in high-income countries.^11^ For instance, people in the United States had fewer strokes, and were less likely to die after strokes, in 2011 than in 1987.^12^ PAHO/World Health Organization (WHO) age-adjusted estimates for cerebrovascular mortality by country in 2013 (except years as indicated) are shown in Figure 1.^10^ The GBD 2013 survey demonstrated that stroke was among the ten leading causes of disability-adjusted life-years (DALYs) in the majority of countries in Latin America and the Latin Caribbean, and was one of the five leading causes in most of them.^11^

Stroke prevalence in Latin America per 1,000 population based on door-to-door surveys ranges from 1.7 among rural Bolivians to 7.7 among urban Mexicans.^12^^,^^13^^,^^14^In the PISCIS (Proyecto Investigación de Stroke en Chile: Iquique Stroke) study among a predominantly Hispano-Mestizo population, the age-adjusted incidence of first-ever stroke was 1.40 per 1,000 (95% confidence interval, 1.24, 1.56).^15^ Among individuals aged > 60 years, the crude prevalence of stroke ranges from 18.2 per 1,000 in Mexico to 46.7 per 1,000 in Colombia,^13^ in line with the observed worldwide trend for increased stroke risk with increasing age.

The leading risk factors for ischemic stroke worldwide are hypertension, smoking, sedentary lifestyle, diabetes and atrial fibrillation.^13^^,^^16^ Although detected less easily than the other risk factors, atrial fibrillation is responsible for 20% of ischemic strokes,^16^ and the one-year mortality risk for Latin American patients with atrial fibrillation is almost twice that found in high-income countries.^17^ Although data on the prevalence of atrial fibrillation in Latin America are limited, a substantial number of people are believed to have arrhythmia. A recent study of atrial fibrillation-related disease and mortality in adults aged > 40 years estimated atrial fibrillation prevalence in the seven countries surveyed as follows: Argentina, 1.95%; Brazil, 1.44%; Chile, 1.68%; Colombia, 1.59%; Mexico, 1.58%; Peru, 1.55%; and Venezuela, 1.47%.^18^ Atrial fibrillation is more common with increasing age: 75% of individuals with atrial fibrillation are aged ≥ 60 years.^18^ Further, many cases of atrial fibrillation are not detected, and there is a clear need to improve the diagnosing of atrial fibrillation so as to reduce stroke risk in Latin American countries.

## OBJECTIVES

Stroke is common but has been incompletely characterized across Latin America. An English-language literature review was conducted to identify the incidence and prevalence of stroke, the approaches to its management and prevention and patient outcomes across a range of countries in Latin America and the Caribbean, with particular attention to the association between stroke and nonvalvular atrial fibrillation, which is an important and underdiagnosed risk factor.

## METHODS

**Figure 2:**
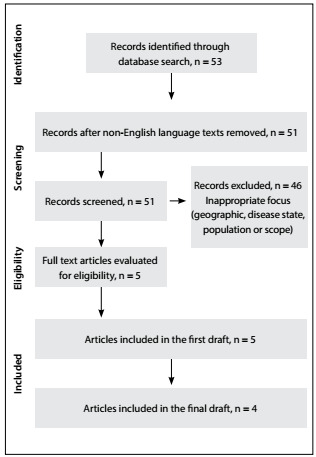
Flow diagram of literature search and disposition.

Information for this narrative review was obtained through a systematic search of the literature to identify published English language scientific papers relating to the search terms. The search terms used were stroke AND "Latin America" AND epidemiology, covering the period from January 2009 to March 2015. The primary search was performed using MEDLINE (via PubMed), Embase (via ProQuest Dialog) and the Cochrane Library. The database search strategy and results are shown in Table 1. Additional searches were conducted in SciELO and in the WHO and PAHO databases to address specific points regarding epidemiology, risk factors and disease management. Reference lists from studies identified through the electronic search were searched manually for further sources. Because the overall yield from PubMed searches was sparse, the authors expanded on the search results, by making further individual searches of relevant publications from January 2000 to March 2015. A flow diagram of the literature search and disposition of the initial structured search is shown in Figure 2. The results from the pivotal trials that demonstrated the efficacy and safety of four non-vitamin K antagonist oral anticoagulants that have been indicated for reducing the risk of stroke in patients with atrial fibrillation are summarized in Table 2.^19^^,^^20^^,^^21^^,^^22^^,^^23^

**Table 1:**
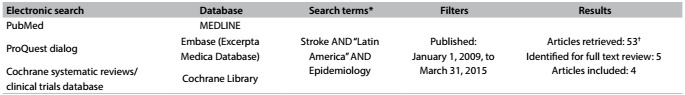
Database search strategy and results

*Search terms were included as MeSH terms when searching MEDLINE and as Emtree terms when searching Embase. In querying the Cochrane library, the search was performed against the title, abstract and key words in the clinical trials and systematic reviews databases; ^†^Excluding cross-database duplicates.

**Table 2:**
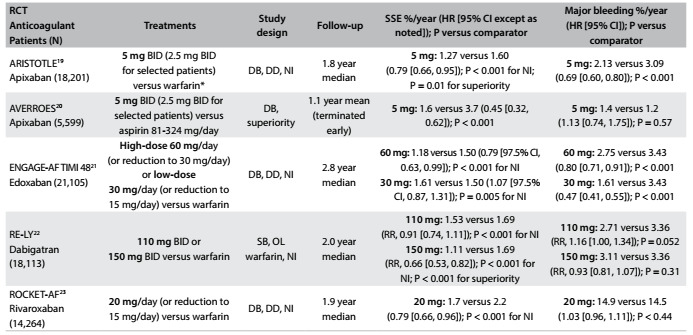
Non-vitamin K antagonist oral anticoagulants: key findings of pivotal randomized controlled trials

Warfarin dose adjusted to target INR 2.0-3.0. BID = twice daily; CI = confidence interval; DB = double-blind; DD = double-dummy; HR = hazard ratio; INR = international normalized ratio; NI = non-inferiority; OL = open-label; RCT = randomized controlled trial; RR = relative risk; SB = single-blind; SSE = stroke or systemic embolism*.*

## RESULTS

### Stroke in Latin America

The populations of Latin America encompass wide ethnic, socioeconomic and geographic variations, and direct comparisons may be misleading. Many factors confound assessments of epidemiology, prevention and treatment. In 1990, the authors of the first global and regional comparative assessment of mortality and DALYs attributable to 10 major risk factors cautioned that different methods of epidemiological assessment for various risks limited the comparability of results.^24^ However, with the development of a common framework and methods, subsequent surveys have afforded opportunities to reassess the evidence for exposure and effect sizes for a much broader set of risk factors.^11^

According to WHO, the estimated stroke mortality per 100,000 population in 2008 was 11.3 for Latin Americans aged ≤ 60 years, compared with 301.3 for those aged 61-79 years.^25^ International differences in case-fatality rates and the proportions of patients who have died or remain dependent six months after a stroke have been attributed to differences in acute care, including access to stroke units and computed tomography scans, and in aspirin use on discharge.^6^

Stroke-associated costs are substantial. National expenditure for ischemic stroke management in 2008 was US$ 326.9 and 239.9 million in Brazil and Argentina, respectively.^26^^,^^27^ The mean per-patient cost of hospitalization for ischemic stroke was $1,902 in Brazil versus $3,888 in Argentina for a similar mean duration of hospital stay: 13.3 and 13.0 days, respectively.^26^^,^^27^ The personal financial burden can be heavy for Latin Americans, many of whom incur high out-of-pocket healthcare costs.^28^

### Health transitions

As the global threat of communicable diseases recedes, chronic and non-communicable conditions are taking their place.^13^^,^^29^ For Brazil overall, this shift occurred in the 1960s, but analysis confined to the major cities shows that cerebrovascular mortality rates began to exceed mortality from other cardiovascular conditions, such as rheumatic heart disease and syphilitic aortic disease, as early as the mid-1940s.^30^ The GBD study in 2013 found that the leading risk factors threatening global health were those underlying non-communicable diseases, including stroke: high blood pressure, smoking, diet, obesity, elevated blood glucose, dyslipidemia, air pollution, and alcohol over-consumption.^13^ Furthermore, Latin America is undergoing a transition in which, although deaths from chronic diseases now exceed deaths from infections and malnutrition for most of the region, residents of underserved regions still remain at risk of infections and malnutrition, which are associated with an elevated risk of stroke.^3^^,^^6^^,^^13^ Hypertension is acknowledged to be the leading risk factor for stroke in Latin America,^2^^,^^11^ but because hypertension and other preventable risk factors are amply covered in the literature, the present review focused on atrial fibrillation, a less easily diagnosed but significant and treatable cause of ischemic stroke.

### Poverty: a global risk factor for stroke

The Latin American/Caribbean region, like many others, has considerable economic inequality and a widening income gap.^6^^,^^29^ Stroke prevalence rates are higher in poorer areas than in more affluent areas. In a 2010 survey of low-income residents in São Paulo aged > 35 years, 5.4% self-reported prior stroke. The age-adjusted prevalence rates for men and women were 4.6% and 6.5%, respectively, i.e. higher than those reported in many other countries.^31^ Another study on low-income residents aged > 65 years in São Paulo found higher prevalence of coronary heart disease, left bundle-branch block and atrial fibrillation, consistent with the burden of stroke mortality in Brazil.^32^ However, this disparity is not limited to urban areas, given that a 2011 survey of residents aged > 35 years in *ribeirinha* communities compared with urban residents of the same municipality found higher crude prevalence of stroke in rural areas (6.3 versus 3.7%, respectively) after adjustment for sex and age.^33^ Despite trends showing declining stroke mortality rates across Brazil consistent with the worldwide trend, stroke mortality rates remain high among low-income Brazilians and have not dramatically decreased over the last three decades. Reductions in stroke mortality over the last 20 years were greatest in the two wealthiest regions and least in the poorest regions.^34^

The burden of stroke risk factors, and in particular, hypertension, has been characterized as a partial consequence of social determinants, including socioeconomic inequality and perceived discrimination. These are structural factors underlying global health inequalities that exceed the expected influence of access to health services.^30^^,^^35^^,^^36^ Efforts to modify risk factors and alleviate the burden of stroke could be aided by economic and social improvements, including implementation of cost-effective public health policies. Enhanced surveillance efforts in outlier regions where there is a high stroke burden, particularly at low- and middle-income levels, could help to clarify factors implicated in the disproportionate stroke burden and guide interventions with specific goals.^6^

### Atrial fibrillation

The principal mechanism for stroke in patients with atrial fibrillation is embolization of stasis-induced thrombi in the left atrial appendage. The risk of stroke in patients with atrial fibrillation increases with age and other risk factors, including hypertension, diabetes, heart failure and previous stroke.^16^^,^^37^ Strokes associated with atrial fibrillation are generally more severe, cause greater disability and are associated with worse outcomes than are strokes in patients without atrial fibrillation.^37^ In a survey of seven countries in Latin America, more than half of the patients with atrial fibrillation were receiving medical treatment, but a significant proportion of the 60% treated as outpatients were not receiving appropriate anticoagulant therapy, despite having a high stroke risk.^18^ Moreover, the proportion of patients with atrial fibrillation receiving treatment within the national healthcare system decreased with increasing age across all countries. Cost and lack of health infrastructure are major barriers to care, and suboptimal care is associated with poor outcomes.^1^^,^^3^^,^^6^ The Mexican PREMIER registry investigators urged secondary prevention to modify stroke risk factors, including atrial fibrillation.^38^

### Guidelines and management initiatives

Patients with atrial fibrillation are an important target for efforts to reduce the risk of stroke through anticoagulant therapy. Vitamin K antagonists, such as warfarin, phenprocoumon and acenocoumarol, are widely prescribed in Latin America. Compared with no therapy, vitamin K antagonists reduce the risk of stroke by 62-68% and the rate of death by 26-33%.^37^ For every 1,000 patients adequately treated with warfarin, 31 ischemic strokes are prevented each year.^39^

Globally, fewer than half of patients with atrial fibrillation receive adequate anticoagulation to reduce the risk of embolic stroke.^40^ The Acute Decompensated Heart Failure National Registry (ADHERE), which enrolled patients with decompensated heart failure and either new-onset or a history of atrial fibrillation in 10 countries across Latin America/Asia-Pacific, found that prophylactic anticoagulation was underused, with significant differences in use among the participating countries.^41^ Many physicians may overestimate bleeding risk and underestimate the benefits of stroke prevention measures.^40^^,^^41^ The investigators noted misuse of anticoagulant therapy, with greater warfarin use among patients with low stroke risk, according to a validated screening tool, and little warfarin use among high-risk patients in greatest need of anticoagulation.^40^ The Latin American Society of Cerebrovascular Diseases advises warfarin for patients with a moderate-to-high risk of stroke, as recommended by the European Society of Cardiology 2010 guidelines.^42^^,^^43^ However, the well-known complexities of warfarin therapy may hinder its use. Warfarin requires frequent monitoring of anticoagulant effect, dose adjustments and close attention to diet. Difficulties of access to monitoring, including distance and cost, may help explain why physicians hesitate to prescribe warfarin for patients with limited resources.

After the Iberoamerican Society of Neurology declared stroke a catastrophic disease in 2004,^44^ medical specialists organized to improve the quality of care. Public health programs promoting national stroke days and other efforts to raise awareness have been established throughout Latin America.^6^ Several countries have participated in the WHO STEPwise approach to Surveillance (STEPS) stroke program to standardize stroke data, estimate the resources necessary for preventing stroke and measure the effects of public health efforts.^6^^,^^45^

Since 2006, the Chilean Ministry of Health national guidelines have guaranteed a minimum level of care for every patient with ischemic stroke, with assurance of rapid neurological assessment, computed tomography scans, hospitalization, neurorehabilitation and secondary prevention.^6^ At a Cuban stroke center, a 10-component fast-track approach doubled hospital admission rates and halved case-fatality rates in the region from 1990 to 2003.^46^ Many hospitals have established stroke units, which increase the likelihood of good outcomes, although stroke units are yet to be adopted as national health care policy by any regional government.^6^ Practices at a comprehensive care center in Brazil changed following a retrospective study showing that adequate oral anticoagulation for patients with atrial fibrillation could have prevented half of all strokes.^47^ However, another survey at a tertiary care clinic in São Paulo showed that only 55% of patients with atrial fibrillation and high stroke risk received dose-adjusted warfarin.^48^ In a Mexican cohort, fewer than half of patients with nonvalvular atrial fibrillation and cerebral infarction were discharged with an oral anticoagulant; patients who lived in rural areas or had functional impairment on discharge were least likely to be prescribed warfarin.^49^

In October 2015, in support of the WHO 25/25 goal of achieving a 25% decrease in premature mortality due to non-communicable diseases by 2025, representatives of government institutions, scientific and professional societies, academic institutions and health policy bodies across Latin America issued a unified call for action regarding prevention and treatment of stroke in the Americas.^50^ The Declaration of Santiago de Chile (Scaling up Stroke Prevention and Treatment in the Americas) urged regional authorities to allocate financial and human resources commensurate with local and regional stroke burdens.^50^ It stressed the need to prioritize strategies within national and regional institutions to achieve organized systems of stroke care and emphasized the importance of primary and secondary prevention, including pharmacological management of treatable risk factors by means of antihypertensives, traditional and newer anticoagulants in patients with atrial fibrillation, lipid control therapies and antiplatelet therapy.^50^

### Non-vitamin K antagonists for stroke prevention in nonvalvular atrial fibrillation

Treatment with newer oral anticoagulants, which are given as fixed doses, do not require monitoring, have predictable clinical effects and have better safety profiles than vitamin K antagonists. They may improve preventive care for patients with nonvalvular atrial fibrillation who are at risk of stroke, in regions with limited access to medical resources. Non-vitamin K antagonist oral anticoagulants were approved for reducing the risk of stroke in patients with nonvalvular atrial fibrillation after large international trials demonstrated their efficacy and safety versus warfarin (Table 2).^19^^,^^20^^,^^21^^,^^22^^,^^23^The agents that have been approved are dabigatran, rivaroxaban and apixaban. Edoxaban, recently approved in the United States, may soon be approved in Latin America. Latin American tertiary centers contributed approximately 20% of the participants in the clinical trials.^19^^,^^20^^,^^21^^,^^22^^,^^23^

The Randomized Evaluation of Long-Term Anticoagulation Therapy (RE-LY) study compared dabigatran (150 mg or 110 mg twice daily) with adjusted-dose warfarin in more than 18,000 patients with nonvalvular atrial fibrillation who were at moderate-to-high risk of stroke or systemic embolism, including 1,134 patients from Latin American tertiary centers.^22^ The ROCKET-AF (Rivaroxaban Once Daily Oral Direct Factor Xa Inhibition Compared with Vitamin K Antagonism for Prevention of Stroke and Embolism Trial in Atrial Fibrillation) study on more than 14,000 patients with nonvalvular atrial fibrillation included 1,878 patients from Latin America in the intention-to-treat population.^23^ The ARISTOTLE (Apixaban for Reduction in Stroke and Other Thromboembolic Events in Atrial Fibrillation) trial assessed the efficacy and safety of apixaban (5 mg twice daily; 2.5 mg for selected patients) in more than 18,000 patients with nonvalvular atrial fibrillation and ≥ 1 additional risk factor for stroke, including 3,468 patients from Latin America.^19^ The AVERROES (Apixaban Versus Acetylsalicylic Acid to Prevent Stroke in Atrial Fibrillation Patients Who Have Failed or Are Unsuitable for Vitamin K Antagonist Treatment) study, a comparison of apixaban with aspirin (81-324 mg daily), included 5,599 patients, of whom 1,185 were from Latin America.^20^ In the ENGAGE AF-TIMI 48 (Effective Anticoagulation with Factor Xa Next Generation in Atrial Fibrillation-Thrombolysis in Myocardial Infarction 48) trial, there were more than 21,000 patients with nonvalvular atrial fibrillation, including 2,661 from Latin America.^23^ In the pivotal trials, primary efficacy and safety findings were consistent across subgroups, including those in Latin America and other geographic regions worldwide.^19^^,^^20^^,^^21^^,^^22^^,^^23^

Non-vitamin K antagonist oral anticoagulants are easier to use than warfarin, are at least as effective and are associated with lower rates of intracranial hemorrhage.^19^^,^^20^^,^^21^^,^^22^^,^^23^Acquisition costs for these drugs are higher than for warfarin, but a cost-benefit review of treatment for atrial fibrillation, based on observational studies, suggested that the overall cost of therapy may be lower because, in contrast to warfarin, dose adjustment and routine monitoring of the anticoagulant effect are not required, and the risk of complications from therapy over the long term may be lower.^51^ However, cost-effectiveness comparisons of non-vitamin K antagonist agents versus warfarin are warranted before the results can be directly applied to the real-world setting.^51^

Observational studies and registries have begun assessing the impact, safety and efficacy of non-vitamin K antagonist oral anticoagulants for reducing stroke risk in cases of nonvalvular atrial fibrillation in routine clinical practice around the world. GLORIA-AF (Global Registry on Long-Term Oral Antithrombotic Treatment in Patients with Atrial Fibrillation, NCT01428765) is a multinational, prospective registry designed to characterize the treatment of patients newly diagnosed with nonvalvular atrial fibrillation who are at risk of stroke and are receiving treatment with warfarin, aspirin or non-vitamin K antagonist oral anticoagulants. XANTUS-EL (Xarelto for Prevention of Stroke in Patients with Nonvalvular Atrial Fibrillation, Eastern Europe, Middle East, Africa and Latin America, NCT01800006) is evaluating the real-world use of rivaroxaban. The PINNACLE (Practice Innovation and Clinical Excellence) registry and research alliance (an outpatient cardiology registry) is calculating performance measurements for outpatient management of several cardiovascular conditions, including atrial fibrillation, in the United States and other countries. These registries will provide important real-world information on anticoagulant prescribing patterns and outcomes.

## DISCUSSION

Shifts in risk factors, economic and social influences and health effects in Latin America have exposed stroke and its consequences as a serious public health problem, which was described as catastrophic a decade ago. Studies currently in progress will build a base of evidence for management and prevention of stroke across Latin America. Today, stroke mortality in the region remains higher than in the developed world, but stroke mortality rates in Latin America have declined, especially in wealthier regions, and this trend could continue if countries were to gain control over a number of modifiable cardiovascular risk factors while implementing public health measures to continue with improvement to social and economic conditions. Patients with nonvalvular atrial fibrillation are an important population to target in efforts to reduce the burden of stroke across the region. Anticoagulant therapy, appropriately used and monitored, lowers stroke risk among patients with nonvalvular atrial fibrillation by at least two-thirds and mortality by around one-third.^37^ However, anticoagulation is underused owing at least in part to the well-known limitations of vitamin K antagonists. Non-vitamin K antagonist oral anticoagulants may have advantages over vitamin K antagonists, and could play an important role in reducing the risk of stroke among Latin American patients with nonvalvular atrial fibrillation.

## CONCLUSIONS

This narrative review draws on the current literature, including systematic reviews by several investigators, and thus does not report original findings or any results from systematic analysis. Nonetheless, the consensus from this review of the literature indicates that greater awareness and further studies, resources and actions are needed to reduce the heavy and growing burden of stroke in Latin America.
